# Development of Fulminant Type 1 Diabetes Mellitus in a Patient with DRESS Syndrome

**DOI:** 10.1155/2020/9018147

**Published:** 2020-08-30

**Authors:** Pedro Perez, Wilson Sze, Daniel Lozeau, Dipa Avichal, Joshua Miller

**Affiliations:** ^1^School of Medicine, Renaissance School of Medicine at Stony Brook University, 101 Nicolls Road, Health Sciences Center, Level 4, Stony Brook, NY 11794, USA; ^2^Department of Internal Medicine, Endocrinology & Metabolism, Renaissance School of Medicine at Stony Brook University, 101 Nicolls Road, Health Sciences Center, Level 4, Stony Brook, NY 11794, USA; ^3^Departments of Dermatology and Pathology, Renaissance School of Medicine at Stony Brook University, 101 Nicolls Road, Health Sciences Center, Level 4, Stony Brook, NY 11794, USA

## Abstract

Drug reaction with eosinophilia and systemic symptoms (DRESS) syndrome, also known as drug-induced hypersensitivity syndrome, is a serious, sometimes lethal, immunological reaction to drug metabolites involving multiple organ systems. Some of the common causative agents of DRESS include allopurinol, minocycline, sulfasalazine, azathioprine, antiepileptic drugs, and hydroxychloroquine. DRESS is often misdiagnosed and challenging to clinically manage due to the disease's myriad presentations, acute complications, and long-term sequela after initial resolution. We present the case of a 39-year-old female patient that developed type 1 diabetes as a sequela of DRESS. The patient originally presented to the emergency department with three days of fevers and a pruritic erythematous maculopapular rash that began two weeks prior. She had recently started an antibiotic course and had also been on a long-term antiepileptic drug regimen. Following a thorough clinical examination, the patient was diagnosed with DRESS and treated accordingly. Over the next four months, she went on to have multiple hospitalizations with several admissions to the medical intensive care unit. She had numerous complications including significant facial edema, seizures, bacterial pneumonia, sepsis, hypovolemic shock, acute respiratory distress syndrome, diabetic ketoacidosis, nonalcoholic steatohepatitis, liver failure, and recurring DRESS rashes despite treatment with high-dose intravenous steroids and immunosuppressants. During this time, the patient developed a rare form of uncontrolled type 1 diabetes mellitus not explained by autoantibody production or continued high-dose steroid use. Fulminant type 1 diabetes mellitus is a sequela of DRESS that is poorly understood and rarely reported. When it occurs, it significantly and negatively affects patient prognosis and requires careful monitoring to assure proper glycemic control.

## 1. Introduction

Drug reaction with eosinophilia and systemic symptoms (DRESS) syndrome, also known as drug-induced hypersensitivity syndrome (DIHS), is a serious, sometimes lethal, immunological reaction to drug metabolites involving multiple organ systems [1]. DRESS falls within a spectrum of skin and mucous membrane drug reactions termed severe cutaneous adverse reactions (SCARs), which contains other pathologies such as Steven–Johnson syndrome and acute generalized exanthematous pustulosis (AGEP). DRESS is typically characterized by fevers, facial edema, erupting skin rash, eosinophilia, atypical lymphocytosis, and lymphadenopathy [2]. The liver is often the primary visceral organ affected; however, other organs, such as the spleen, can also be involved [3]. There are reported autoimmune sequelae of DRESS such as systemic lupus erythematosus, thyroiditis, and diabetes mellitus (DM) [4–7]. Diagnosis of these sequelae is often delayed due to the variable nature of DRESS presentation. Having a clearer understanding of the clinical facets of DRESS will be beneficial in providing rapid identification and treatment, while consequently improving outcomes. In this case report, we present a patient with DRESS that developed a rare form of uncontrolled type 1 DM not explained by autoantibody production or continued high-dose steroid use.

## 2. Case Presentation

**Figure 1:**
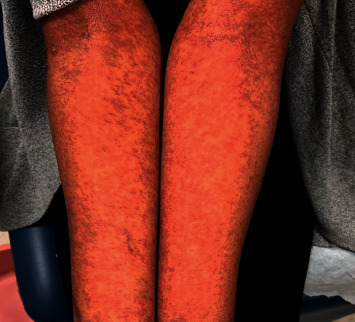
Patient's forearms four weeks following the original erupting rash. Pictured here are follicular erythematous papules with scattered postinflammatory hyperpigmented macules becoming confluent in areas with desquamation. This pattern persisted bilaterally in the trunk and upper and lower extremities.

A 39-year-old African American female with a medical history of chronic focal epilepsy presented to Stony Brook University Hospital emergency department with three days of fevers (>38°C) and mildly pruritic erythematous maculopapular rash. The rash began two weeks prior in the left arm and gradually spread bilaterally to the trunk, face, oropharynx, periocular space, and legs (Figure 1). As there was initial suspicion for a drug-related rash, the patient's medication history was reviewed. She had been recently started on an antibiotic course of amoxicillin by her primary doctor for unilateral neck lymphadenopathy and exudative pharyngitis. Additionally, she had been on a long-term carbamazepine therapy for epilepsy. One year ago, the patient's antiepileptic medication regimen was changed as she wanted to become pregnant. She had been slowly tapering off carbamazepine and was started on lamotrigine. At the time of presentation, she was taking both carbamazepine and lamotrigine.

**Figure 2:**
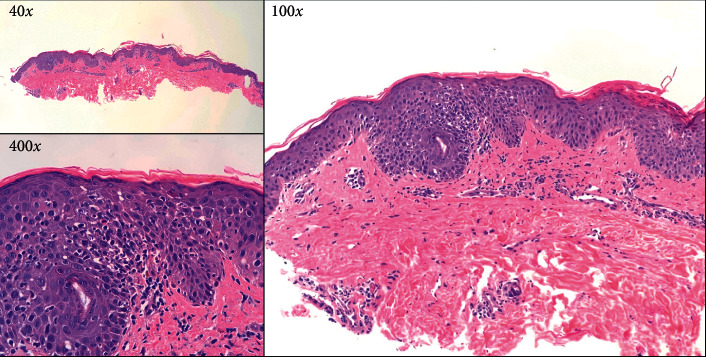
Low-power microscopy images of the patient's skin biopsy (40x and 100x) show interface dermatitis with focal spongiotic dermatitis. On the high-power image, (400x) there is vacuolar interface dermatitis (lymphocytes tagging along the dermoepidermal junction) and rare necrotic keratinocytes within the epidermis. The histopathologic changes are nonspecific but can be observed in drug eruption reactions.

The patient's initial laboratory studies showed an elevated neutrophil count (10.47 K/ul), mild eosinophilia (6–8%), and transaminitis (AST : 48 IU/L; ALT : 42 IU/L). Initial evaluation by the dermatology service suggested a differential diagnosis of AGEP secondary to amoxicillin versus DIHS secondary to lamotrigine or carbamazepine use. A computed tomography (CT) scan revealed no clear etiology of infection, blood cultures were negative, skin cultures from pustules only grew normal skin flora, and skin biopsy revealed nonspecific findings most consistent with AGEP (Figure 2). CT abdominal scan showed multiple wedge-shaped lesions within the spleen suspicious for focal areas of infarction and ischemia. This discovery in conjunction with the rest of the patient's presentation yielded a diagnosis of DRESS [8]. Over the next four months, this patient would have multiple hospitalizations with several admissions to the medical intensive care unit (MICU). The patient had numerous complications including significant facial edema, seizures, bacterial pneumonia, sepsis, hypovolemic shock, acute respiratory distress syndrome (ARDS) requiring intubation, diabetic ketoacidosis (DKA), nonalcoholic steatohepatitis, liver failure requiring transfer to a liver transplant center (transplant was ultimately not needed), and recurring DRESS rashes despite treatment with high-dose intravenous steroids and immunosuppressants (e.g., cyclosporine). Of interest to this case report, the patient developed persistently uncontrolled hyperglycemia complicated by DKA in the setting of sepsis.

Approximately, a month following onset of the rash, the patient's serum glucose was found to be over 500 mg/dL with a hemoglobin A1C of 7.1% without a prior history of diabetes mellitus (DM). The patient was started on insulin therapy for probable steroid-induced diabetes mellitus (SIDM), as she was requiring a high-dose steroid regimen for DRESS. The patient's SIDM was complicated by DKA during a sepsis episode precipitated by a bacterial pneumonia. She remained in the MICU for several weeks where she was managed with respiratory support, antibiotics, insulin, and steroids. She was eventually discharged from the hospital following improvement of her respiratory status, sepsis, liver failure, DRESS rash, and serum glucose levels. The steroids were tapered off during outpatient follow-up, and her SIDM briefly improved. However, despite continuing a steroid taper, she had significant fluctuating fasting glucose levels and her insulin regimen was titrated. The patient continued to have persistently elevated serum glucose levels (>300 mg/dL) requiring insulin therapy despite cessation of steroids. She required multiple daily insulin injections and wore a continuous glucose monitoring device (CGM). Glutamic acid decarboxylase (GAD) antibodies and islet cell cytoplasmic autoantibodies (ICAs) were tested for correlation with autoimmune subtype of type 1 DM; however, they were found to be negative. The patient's new onset uncontrolled type 1 DM was originally diagnosed as SIDM, which normally improves following reduction or cessation of steroids. However, given her worsening glycemic control in the context of tapering and short-term cessation of steroids, the patient was diagnosed as having a rare but previously documented type of fulminant type 1 DM as a sequela of DRESS [4].

## 3. Discussion

DRESS syndrome is a clinically significant skin phenomenon with systemic involvement within the SCARs phylogeny of cutaneous immune responses to drug exposure. DRESS can be lethal with a mortality rate approaching 10% and is believed to occur with an incidence ranging from 1 : 1000 to 1 : 10,000 drug exposure, although exact incidence is unknown and may vary by population [2–4]. DRESS, which is ultimately a clinical diagnosis, can often be misdiagnosed as well as mismanaged due to its varying presentations, unique and difficult manifestations, and hosts of sequelae. The patient presented here developed fulminant type I DM as a sequela of DRESS and required increasing doses of insulin and a CGM device. If this patient's DM continues to progress, they may require an insulin pump, thus highlighting the serious nature of this DRESS sequela.

The patient's early presentation was classic of DRESS syndrome, with lymphadenopathy, facial edema, erupting morbilliform rash, fevers, and liver and spleen involvement. It is worth noting that she initially had mild transaminitis, which quickly progressed to significant transaminitis and near liver failure; thus, the original liver enzyme values are not necessarily prognostic of disease progression. Nevertheless, liver involvement is highly correlated with DRESS and found to occur as high as 87% of DRESS patients [3]. Moreover, this patient had splenic infarcts, a finding previously appreciated in the literature in DRESS patients [8]. There are multiple published criteria that are used for diagnosing DRESS. Among these, RegiSCAR and particularly Bocquet's criteria have been found to be diagnostically superior [9]. The seven characteristics (three of seven characteristics must be met) of the RegiSCAR criteria are skin eruption, fever (>38°C), lymphadenopathy at two or more sites, involvement of one internal organ, lymphocytosis or lymphopenia, blood eosinophilia (>10% or 700/*µ*L), and thrombocytopenia (<120 × 10^3^/*µ*L). Alternatively, simpler Bocquet's criteria require skin eruption, blood eosinophilia (>1.5 × 10^3^/*µ*L), and internal organ involvement. Under these guidelines, this patient definitively meets four of the seven RegiSCAR characteristics and closely approaches one of the remaining three (mild eosinophilia below criteria threshold). Likewise, the patient meets two of the three characteristics of Bocquet's criteria with eosinophil levels below the suggested threshold. The patient's eosinophil levels were not closely monitored after admission, thus raising the possibility that the levels may have increased later during disease progression.

It is not fully clear what agent induced DRESS syndrome in this case presentation. The patient had been on long-term carbamazepine treatment for epilepsy, had begun lamotrigine a few months prior, and just initiated a course of amoxicillin. *β*-lactams are more frequently associated with AGEP; however, they are also known to cause DRESS flare-ups in patients with DRESS induced by a different drug type [10, 11]. Anticonvulsants are well-recognized for inducing DRESS, particularly lamotrigine and carbamazepine [2, 3]. However, this patient had been on carbamazepine for over ten years and had been tapering it over the last year. This makes carbamazepine an unlikely trigger of DRESS in this scenario. The patient's DRESS rash had been ongoing for about two weeks before presentation. She began taking amoxicillin a few days before needing to come to the hospital. As DRESS normally takes 2–12 weeks to develop after drug exposure, it seems plausible that lamotrigine may have originated the DRESS syndrome, and this was exacerbated by amoxicillin use.

The patient's hyperglycemia was first noted in outpatient setting about a month following the original DRESS rash. As the patient had been on an intensive steroid and immunosuppressant treatment regimen for DRESS, the globally elevated hyperglycemia was believed to be SIDM. SIDM is an expected outcome of long-term medical glucocorticoid use. The prognosis for SIDM is generally positive; once the steroid dosing is reduced, insulin requirements decrease, and normal endocrine function is reestablished [12]. While in the hospital, the patient continued to receive insulin to control her serum glucose level. However, once the DRESS syndrome improved and she was tapered from steroids, her serum glucose levels paradoxically continued to increase. This persisted for months and required close endocrinology outpatient follow-up. Past studies have reported thyroiditis, systemic lupus erythematosus, autoimmune hemolytic anemia, vitiligo, alopecia, and type 1 DM as autoimmune sequelae of DRESS [5, 6, 13, 14]. Previously reported case reports of type 1 DM as sequela of DRESS were characterized by sudden onset typically between 2 weeks−10 months post-DRESS, lack of GAD or ICA antibodies, low or absent C-peptide levels, and significant death of pancreatic *β*-cells. However, there has also been at least one report of GAD-positive post-DRESS type 1 DM [7]. The rapid onset and lack of pancreatic autoantibodies are more consistent with this patient's presentation; this subtype has been previously termed nonautoimmune fulminant type 1 DM.

Nonautoimmune fulminant type 1 DM as a development from DRESS is uncommon and has been scarcely reported. A previous study by Chen et al. found 1 in 43 patients with fulminant type 1 DM 1-2 months post-DRESS [6]. A similar study by Kano et al. observed 5 in 145 patients having type 1 DM in a similar time frame after DRESS resolution [13]. Finally, Chiou et al. reported 2 in 30 patients with type 1 DM post-DRESS [15]. Interestingly, it was previously found that the MHC I serotype, HLA-B62, was found in a greater frequency in fulminant type 1 DM associated with DRESS syndrome [16]. In a follow-up study, we would like to test the MHC serotype present in this patient. The mechanisms underlying the development of fulminant type 1 DM as a sequela of DRESS are complicated and not well understood. There seems to be an association with reactivation of herpes virus HHV-6, as well as a loss of suppressive function by T-regulatory cells, a type of immunosuppressant T-cell.

Fulminant type 1 DM as a sequela of DRESS is a poorly understood and rarely reported clinical phenomenon. When it occurs, it significantly and negatively affects the prognosis and requires a constant, careful, and financially costly management of these patients to assure proper glycemic control. Earlier identification of this chronic complication of DRESS will aid clinicians in more efficient treatment that will improve patient outcomes. Clearly, more work is needed to further elucidate the physiology underlying this unique pathology.
